# Supplementary material for: Cloud BioLinux: pre-configured and on-demand bioinformatics computing for the genomics community
Source: BMC Bioinformatics. 2012 Mar 19;13:42. doi: 10.1186/1471-2105-13-42 (PMC3372431; doi:10.1186/1471-2105-13-42)
Supplement: Additional file 1 — Supplementary 1 Cloud BioLinux software documentation in the form of a mini, self-contained website. Users need to download and uncompress the .zip file, and open through a web browser the "index.html" file available on the main directory. (ZIP 1823 kb). [file 1471-2105-13-42-S1.ZIP › Cloud-BioLinux-Package-Documentation/docs/caf2gap.html]

Bio-Linux Software Documentation Pages

Back to search form

## caf2gap

|  |  |
| --- | --- |
| Name | caf2gap |
| Description | **caf2gap** is released within the bio-linux-assembly-conversion-tools package.  This script converts assemblies in the common assembly format (caf) to the gap4 assembly format. A related script called gap2caf is also available.  For further details about this script and how to work with it, please see the remote documentation. |
| Homepage | http://www.sanger.ac.uk/Software/formats/CAF/ |
| Remote Documentation | http://www.sanger.ac.uk/Software/formats/CAF/userguide.shtml |
